# Pancreatic stent migration into the main pancreatic duct during endoscopic papillary balloon dilation

**DOI:** 10.1002/deo2.363

**Published:** 2024-04-13

**Authors:** Toji Murabayashi, Mayu Kawabata, Shinya Sugimoto

**Affiliations:** ^1^ Department of Gastroenterology Ise Red Cross Hospital Mie Japan

**Keywords:** EPBD, ERCP, migrate, MPD, removal

## Abstract

We report the first case of pancreatic stent (PS) migration placed early into the main pancreatic duct (MPD) during endoscopic retrograde cholangiopancreatography (ERCP) due to subsequent endoscopic papillary balloon dilation. A 74‐year‐old woman who complained of fever and abdominal pain was diagnosed with acute calculous cholangitis. On ERCP, a needle‐knife precut papillotomy was performed from the orifice because of difficult cannulation. Because of unintentional guidewire insertion into the MPD from the orifice, a PS with bilateral flaps was promptly placed to prevent post‐ERCP pancreatitis. After successful biliary cannulation from the orifice alongside the PS, endoscopic papillary balloon dialtion was performed, leading to PS migration into the MPD during the dilation. Two days after the first ERCP, the migrated PS was successfully removed on the second ERCP. The strategy of early PS placement in the ERCP session appears theoretically promising for preventing post‐ERCP pancreatitis. However, early PS placement during the ERCP session should be noted to pose the risk of migration into the MPD, especially when pushing the device into the bile duct.

## INTRODUCTION

In cases involving difficult biliary cannulation, with ready access to the main pancreatic duct (MPD) during endoscopic retrograde cholangiopancreatography (ERCP), placing a pancreatic stent (PS) is recommended to prevent post‐ERCP pancreatitis.^1^, ^2^, ^3^ While prophylactic PS placement is typically performed later in the ERCP session, evidence suggests it may be more effective when performed early.^4^ The strategy of early PS placement in the ERCP session appears theoretically promising for preventing post‐ERCP pancreatitis. However, there is a potential risk of PS migration into the MPD during subsequent procedures. Herein, we present the first reported case where a PS, inserted early in the session, migrated into the MPD due to subsequent endoscopic papillary balloon dilation (EPBD) for the biliary orifice during ERCP.

## CASE REPORT

**FIGURE 1:**
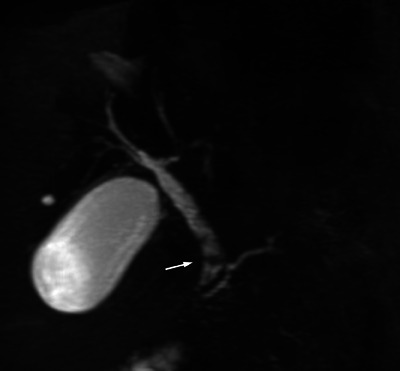
Magnetic resonance cholangiopancreatography image showing a filling defect (arrow) in the bile duct.

**FIGURE 2:**
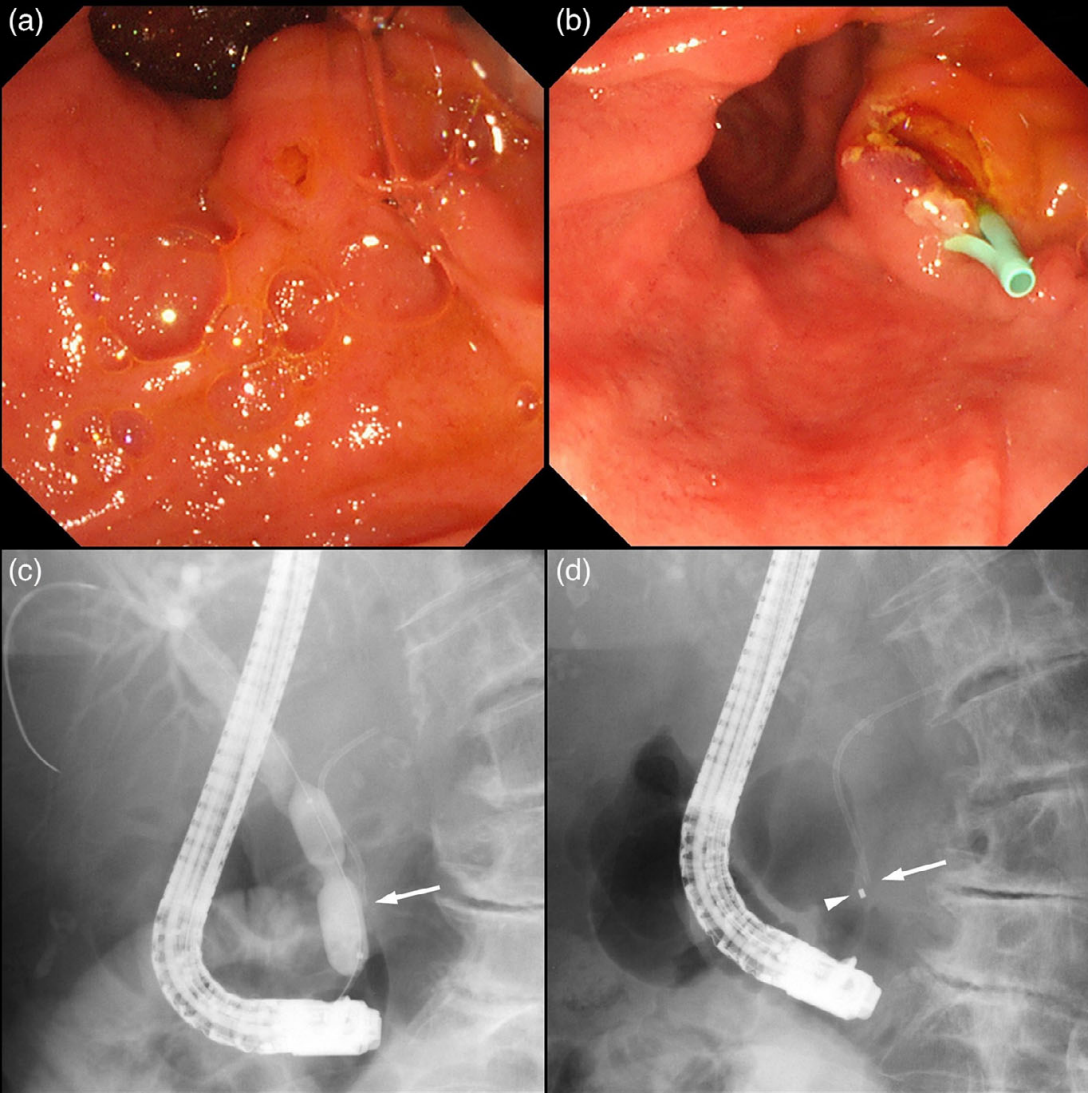
Endoscopic and fluoroscopic images obtained during the first endoscopic retrograde cholangiopancreatography. (a) Endoscopic image showing the papilla of Vater and a large peripapillary diverticulum. (b) Endoscopic image showing the pancreatic stent (PS). The orifice of the papilla is opened by needle‐knife precut papillotomy. (c) Fluoroscopic image obtained during endoscopic papillary balloon dilation for the biliary orifice. The distal edge of the PS before the migration is indicated by an arrow. d: Fluoroscopic image after the migration of the PS into the main pancreatic duct. The distal edge of the PS and catheter tip in the papilla are indicated by an arrow and arrowhead, respectively.

**FIGURE 3:**
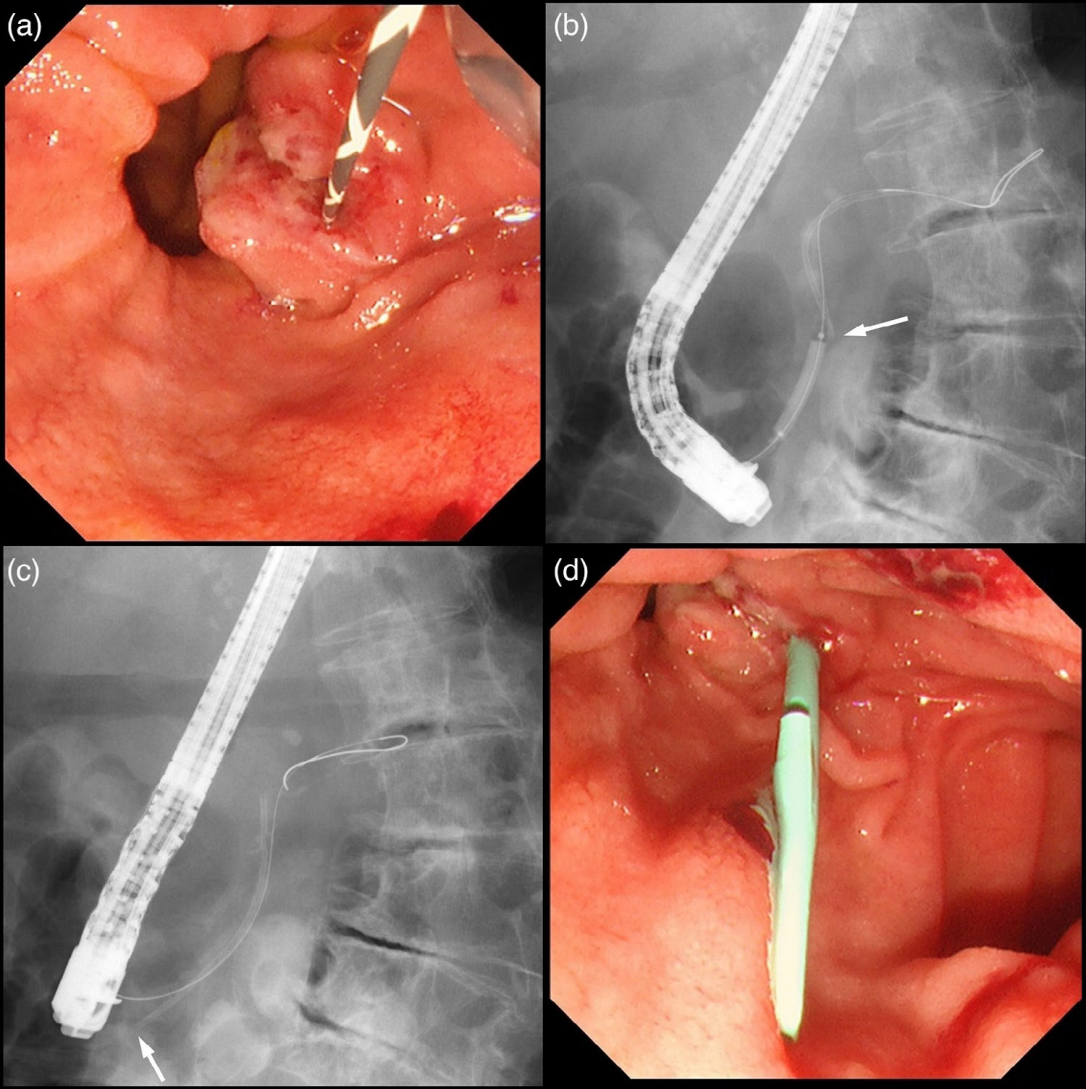
Endoscopic and fluoroscopic images were obtained during the second endoscopic retrograde cholangiopancreatography two days after the first session. (a) Endoscopic image showing the papilla of Vater. The guidewire inserted into the main pancreatic duct is observed, and the distal edge of the pancreatic stent (PS) is invisible. (b) Fluoroscopic image obtained during papillary balloon dilation for the pancreatic orifice. The distal edge of the migrated PS is indicated by an arrow. c: Fluoroscopic image immediately after the successful pull of the PS from the MPD to the duodenum. The distal edge of the PS in the duodenum is indicated by an arrow. d: Endoscopic image showing the PS successfully pulled out from the main pancreatic duct.

A 74‐year‐old woman with no prior history of gastrointestinal disease presented with fever and abdominal pain and was referred to our hospital. Physical examination upon admission revealed the following: blood pressure, 143/78 mmHg; heart rate, 92 beats/min; body temperature, 39.6°C; and mild tenderness in the upper abdomen. Laboratory tests indicated leukocytosis (white blood cell count, 9400 /µL), elevated serum levels of C‐reactive protein (17.1 mg/dL), and hepatobiliary enzymes (aspartate aminotransferase, 723 U/L; alanine aminotransferase, 253 U/L; γ‐glutamyl transpeptidase, 299 U/L; alkaline phosphatase, 208 U/L; total bilirubin, 1.4 mg/dL), with a normal serum amylase level (64 U/L). Computed tomography revealed mild bile duct dilation, a swollen gallbladder, a large peripapillary diverticulum, and no evidence of bile duct obstruction, including common bile duct stones (CBDS). Magnetic resonance cholangiopancreatography confirmed the presence of CBDS (Figure 1), prompting ERCP to address the CBDS causing acute cholangitis (Figure 2). Biliary cannulation using a standard wire‐guided cannulation technique failed due to the large peripapillary diverticulum, and unintentional pancreatic cannulation did not occur. Therefore, a needle‐knife precut papillotomy was performed from the orifice in the upper direction. Because of the unintentional insertion of the guidewire into the MPD from the orifice opened by precut papillotomy during the initial attempt using a wire‐guided cannulation technique, a PS with bilateral flaps (Geenen, 5 Fr × 7 cm; Cook Medical Japan) was promptly placed to prevent post‐ERCP pancreatitis. Biliary cannulation was achieved using wire‐guided cannulation from a well‐opened orifice alongside the PS. As a large peripapillary diverticulum made creating a sufficiently large incision challenging, EPBD using a balloon catheter (Giga II Balloon catheter [10–12 mm]; Century Medical) for the biliary orifice was performed following a small sphincterotomy to facilitate stone removal. Unexpectedly, the PS completely migrated into the MPD while EPBD was performed; this was not noticed during the dilation procedure (Video S1). Despite immediate attempts at pancreatic cannulation via the pancreatic orifice to extract the migrated PS, proper access was hindered by the edematous and peristaltic papillae. The session was completed without removing the migrated PS after successful CBDS removal using basket and balloon catheters. Anticipating spontaneous discharge of the PS into the duodenum within a few days, a follow‐up radiograph 2 days later revealed no evidence of spontaneous discharge. Consequently, a second ERCP was performed to remove the PS that had completely migrated into the MPD (Figure 3). Pancreatic cannulation was easily accomplished using a standard wire‐guided cannulation technique because the edematous papilla had completely improved. Following EPBD using a balloon catheter (REN [4 mm]; Kaneka Medix) for the pancreatic orifice, the PS was removed using a balloon catheter (Extractor ProXL; Boston Scientific Japan) and grasping forceps. Subsequently, the acute cholangitis rapidly improved, and no postoperative adverse events, including post‐ERCP pancreatitis, occurred.

## DISCUSSION

While there have been several reports of PS migration into the MPD after ERCP,^5^, ^6^, ^7^, ^8^ to the best of our knowledge, migration during ERCP has not been previously reported; this is likely because PS placement is typically performed later in ERCP sessions. In the present case, the early placement of the PS in the ERCP session resulted in migration into the MPD following subsequent EPBD for the biliary orifice. The decision to place the PS early during the ERCP session was based on a previous report suggesting that PS placement for preventing post‐ERCP pancreatitis is more effective when performed earlier in the session.^4^ This strategy seems theoretically promising because MPD drainage during subsequent procedures is ensured after PS placement. However, extreme care should be taken when placing the PS early in the ERCP session to avoid migration of the PS during subsequent procedures, especially by biliary insertion of large‐diameter devices, including a balloon catheter for EPBD, as in the present case.

In the present case, two factors potentially increased the likelihood of migration. First, the presence of a large peripapillary diverticulum compromised the endoscopic view of the papilla. Second, the pancreatic duct orifice was dilated using a needle‐knife precut papillotomy. The PS had already moved a little, even after catheter insertion into the bile duct before EPBD, which might have affected the subsequent complete migration by EPBD (Video S1). To reduce the migration risk in such high‐risk groups, using a PS with a pigtail on the duodenal side may be preferable when placing the PS early during the ERCP session. While a straight‐type PS is less likely to interfere with subsequent procedures than a pigtail‐type, the insufficient opening of the flap of the straight‐type PS is less effective in preventing migration, as in the present case. Alternatively, adhering to the conventional practice of placing the PS later during the ERCP session for high‐risk cases can effectively prevent migration.

In conclusion, we report the first documented case of migration of a PS placed early during ERCP into the MPD due to subsequent EPBD for the biliary orifice. It is important to note that early PS placement during the ERCP session poses the risk of migration into the MPD, especially when pushing the device into the bile duct, as in the present case.

## CONFLICT OF INTEREST STATEMENT

None.

## Supporting information


**Video** Endoscopic retrograde cholangiopancreatography during which a pancreatic stent migrated into the main pancreatic duct.
